# Potential Role of Perampanel in Reducing Barbiturate Dependency in Febrile Infection-Related Epilepsy Syndrome: A Case Report

**DOI:** 10.7759/cureus.77568

**Published:** 2025-01-16

**Authors:** Kenta Ochiai, Taku Omata, Kentaro Sano, Hiroshi Sakuma, Jun-ichi Takanashi

**Affiliations:** 1 Department of Pediatrics, Tokyo Women's Medical University Yachiyo Medical Center, Yachiyo, JPN; 2 Department of Brain and Neurosciences, Tokyo Metropolitan Institute of Medical Science, Tokyo, JPN

**Keywords:** barbiturate, febrile infection-related epilepsy syndrome (fires), new onset refractory status epilepticus, paediatric refractory epilepsy, perampanel

## Abstract

Febrile infection-related epilepsy syndrome (FIRES) is a type of new-onset refractory status epilepticus (NORSE) that occurs in previously healthy children. Conventional antiepileptic drugs (AEDs) often fail to control seizures, necessitating the use of high-dose anesthetics, which can lead to severe complications and poor outcomes. Perampanel has shown promise in the treatment of refractory epilepsy; however, its role in FIRES remains underexplored. We report the case of a 13-year-old boy with FIRES, which was characterized by refractory status epilepticus following a febrile illness. Although some AEDs were administered, high-dose thiopental and ventilator support were required. Perampanel at a starting dose of 2 mg/day and titrated to 8 mg/day enabled successful weaning from thiopental and extubation. The patient eventually became seizure-free on clobazam and levetiracetam. On follow-up, the patient exhibited memory and behavioral issues, along with bilateral hippocampal atrophy on MRI. This case demonstrated the potential role of perampanel in managing FIRES by reducing the need for prolonged barbiturate use and ventilator dependence. Although there was persistent cognitive impairment, which was likely secondary to hippocampal damage, perampanel showed a favorable safety profile. This case suggested that perampanel is a valuable addition to FIRES treatment. Further studies are required to confirm its efficacy.

## Introduction

Febrile infection-related epilepsy syndrome (FIRES) is considered a subcategory of new-onset refractory status epilepticus (NORSE) and is characterized by refractory status epilepticus following a febrile illness [1]. The condition predominantly affects previously healthy children and young adults and can lead to significant sequelae and mortality [1]. The exact incidence of NORSE remains unknown; however, the incidence of refractory status epilepticus was estimated to be 3.4-7.2 per 100,000 per year, with NORSE accounting for approximately 20% of these cases [2]. Despite extensive research, the pathogenesis of FIRES remains unclear, and treatment options are limited and often ineffective [3]. Conventional antiepileptic drugs (AEDs) frequently fail to control seizures in FIRES, necessitating the use of high-dose anesthetics to achieve burst suppression on EEG [3]. However, prolonged use of anesthetics is associated with severe complications and can result in poor neurodevelopmental outcomes [4].

In recent years, perampanel, which is an ionotropic noncompetitive antagonist of the alpha-amino-3-hydroxy-5-methyl-4-isoxazolepropionic acid (AMPA) receptor, has emerged as a potential therapeutic option for refractory epilepsy, including cases of FIRES [5]; however, there is limited evidence supporting its use in FIRES. As previously reported, perampanel was initiated in three patients with FIRES. One patient was treated with perampanel at 4 mg/day initially, followed by an increased dose at 10 mg/day, resulting in reduced seizure frequency. The remaining two patients were in a thiopental coma; one of the patients was started on perampanel at 12 mg/day and recovered, whereas the other was started on perampanel at 8 mg/day but showed no improvement in electrographic seizures [5]. This case showed the potential role of perampanel in managing FIRES, as successful weaning from high-dose barbiturate therapy and ventilator support.

## Case presentation

A 13-year-old boy, with no medical history, developed a fever and headache. On the sixth day, he experienced clonic seizures of both upper limbs for several minutes. On the eighth day, the patient was admitted to the hospital because of an hourly cluster of seizures with rightward rotation of the neck and tonic seizures of the right upper limb for several minutes. He was administered midazolam (0.15 mg/kg), fosphenytoin (22.5 mg/kg), and levetiracetam (20 mg/kg); however, the cluster of convulsive seizures did not abate. On the ninth day, he was transferred to our hospital because of difficulties in managing the seizures.

Upon arrival at our hospital, his Glasgow coma scale was E2V3M5 on continuous midazolam at a dose of 0.1 mg/kg/h, and his body temperature was 38.8°C. Blood tests revealed no abnormalities (Table 1).

**Table 1:** Blood test results AST: aspartate aminotransferase, ALT: alanine transaminase, CK: creatine kinase, CRP: C-reactive protein, WBC: white blood cell, Hb: hemoglobin, Plt: platelet, AQP4: aquaporin 4, AFP: α-fetoprotein, HCG-β: human chorionic gonadotropin-β, sIL-2R: soluble interleukin-2 receptor, NSE: neuron-specific enolase

Blood tests			
Test type	Result	Test type	Result
AST	24 U/L	anti-nuclear antibody	(-)
ALT	25 U/L	anti-SS-A antibody	(-)
CK	50 U/L	anti-SS-B antibody	(-)
Ferritin	54.5 ng/mL	anti-AQP4 antibody	(-)
Creatinine	0.52 mg/dL	AFP	1.2 ng/mL
Sodium	141 mEq/L	HCG-β	<1.2 mIU/mL
CRP	0.23 mg/dL	sIL-2R	383 U/mL
WBC	7522 /µL	NSE	16.8 ng/mL
Hb	14.6 g/dL		
Plt	21× 104 /µL		

Cerebrospinal fluid examination showed increased cell count and was negative for bacterial culture, herpes simplex virus polymerase chain reaction, and antineuronal antibodies (Table 2).

**Table 2:** Cerebrospinal fluid examination MOG: myelin oligodendrocyte glycoprotein, NMDAR: N-methyl-D-aspartate receptor, CASPR2: contactin-associated protein-like 2, AMPAR: α-amino-3-hydroxy-5-methyl-4-isoxazolepropionic acid receptor, GABABR: gamma-aminobutyric acid type B receptor, LGI1: leucine-rich, glioma inactivated 1

Cerebrospinal fluid examination	
Test type	Result
Cell counts	41 /µL
Protein	31 mg/dL
Anti-MOG antibody	(-)
Anti-NMDAR antibody	(-)
Anti-CASPR2 antibody	(-)
Anti-AMPAR antibody	(-)
Anti-GABABR antibody	(-)
Anti-LGI1 antibody	(-)

**Figure 1:**
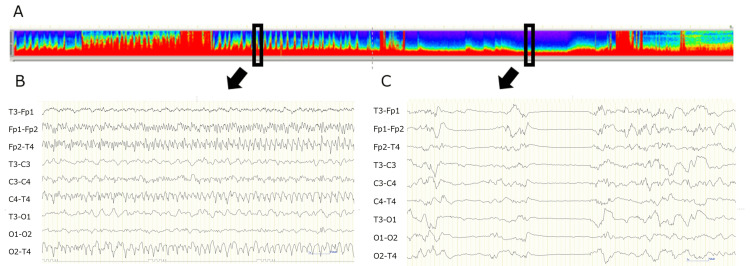
Electroencephalography on day 16 (A) Density-modulated spectral array maps showing high- and low-frequency peaks (in rectangles) every 5 min. (B) Ictal EEG of a high-frequency peak portion (arrow) showing a cluster of apneic seizures during continuous thiopental administration at 4 mg/kg/h. (C) Postictal EEG of a low-frequency peak portion (arrow) during continuous thiopental administration at 8 mg/kg/h.

He was diagnosed with acute encephalitis and started on methylprednisolone pulse therapy (1,000 mg/day for three days). On the 11th day, he experienced a cluster of apneic seizures and was intubated. The seizures were controlled with continuous thiopental administration, and the treatment was later switched to continuous midazolam. However, on the 16th day, thiopental was restarted because of the recurrence of a cluster of apneic seizures. The EEG showed rhythmic waves with frontal dominance approximately every five minutes; the seizures finally resolved after the dose of thiopental was increased to 8 mg/kg/h (Figure 1).

**Figure 2:**
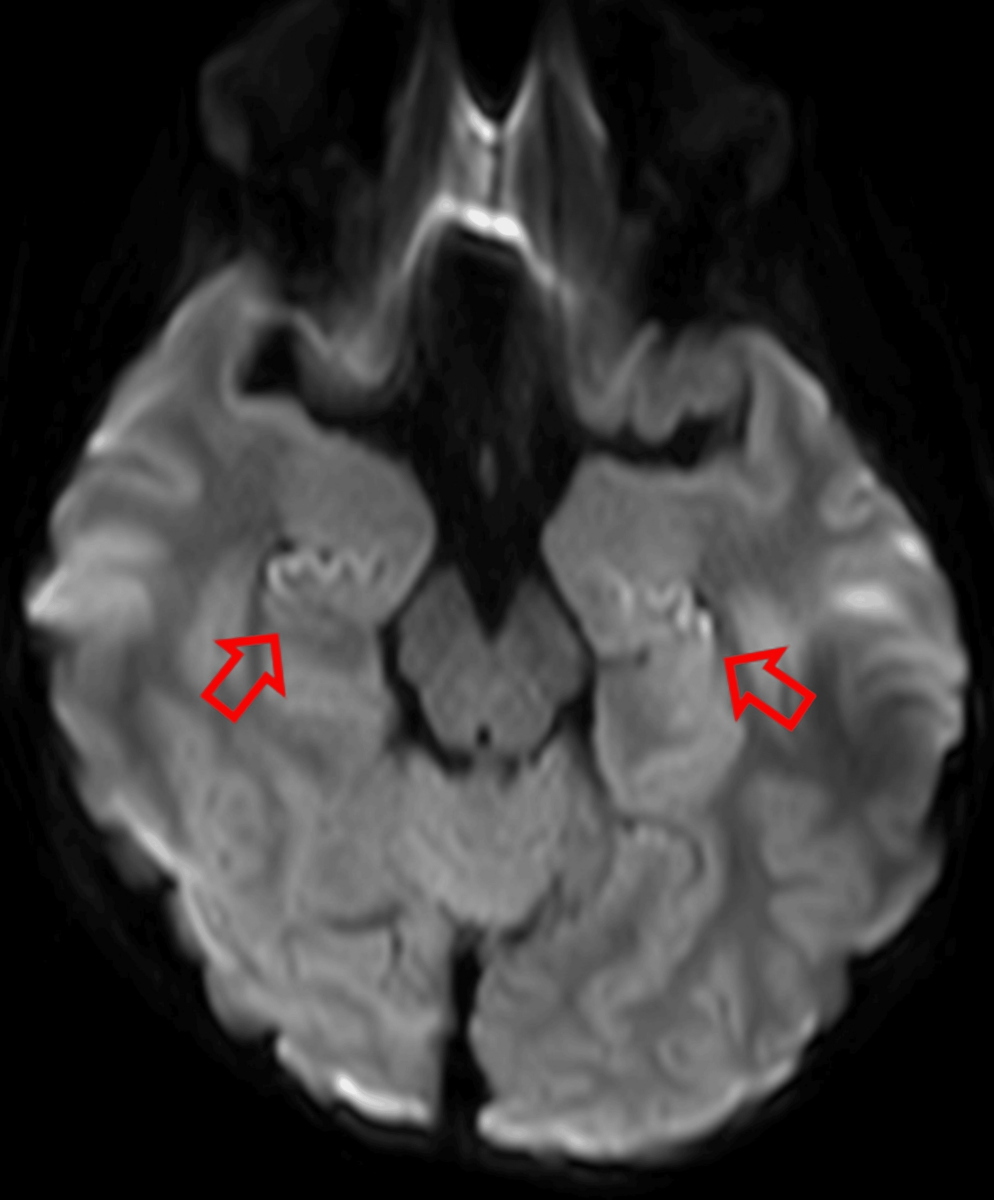
Brain magnetic resonance imaging on day 11 Diffusion-weighted imaging showing hyperintensity in the bilateral hippocampus (red arrows).

MRI showed reduced diffusion in the bilateral hippocampus (Figure 2).

**Figure 3:**
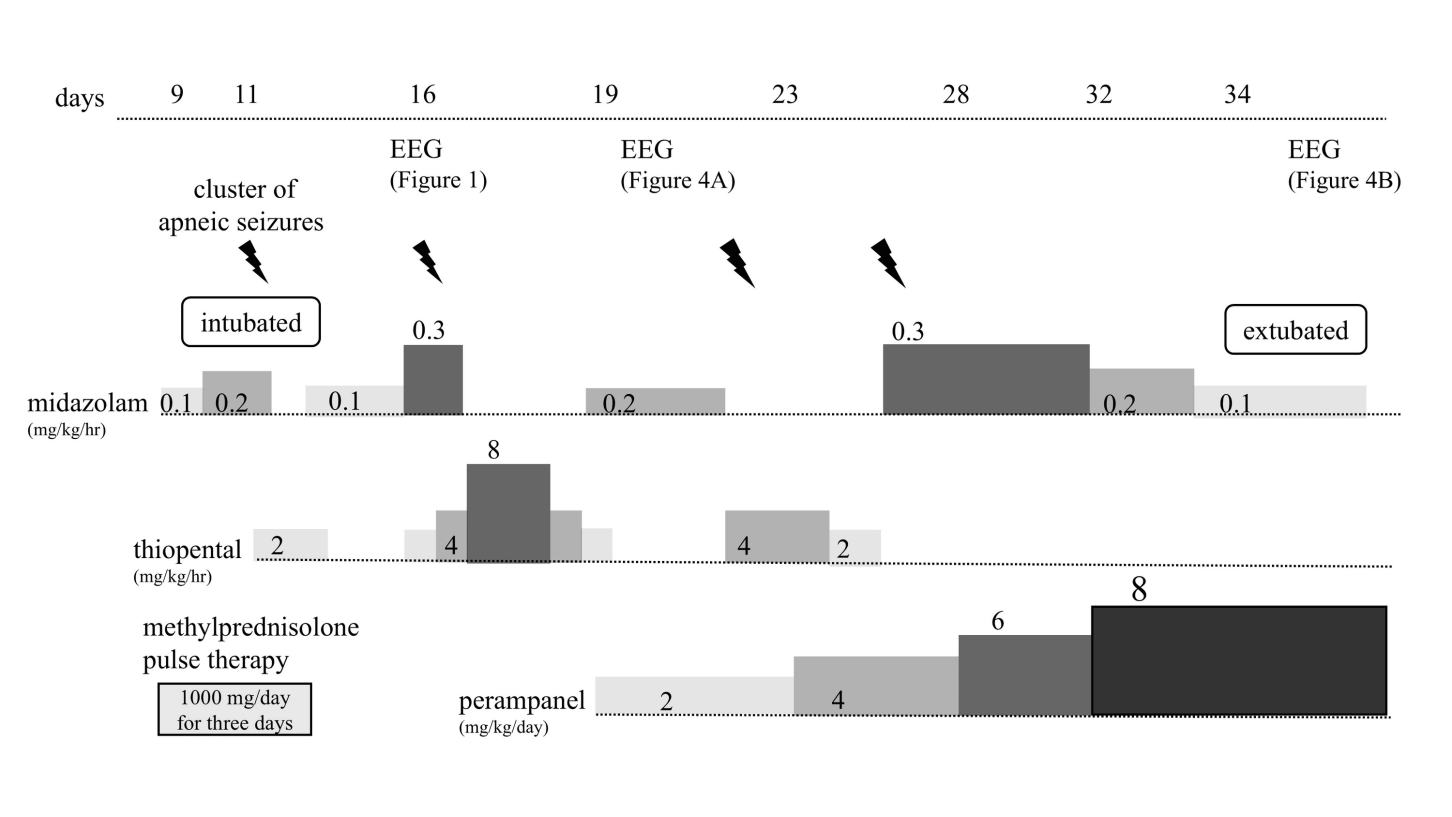
Patient disease course Chart presenting the progress from hospital admission to the start of intensive care management and until extubation.

Because the seizures flared up when the thiopental was tapered, perampanel was started at 2 mg/day and titrated up to 8 mg/day. On the 34th day, thiopental was tapered and changed to continuous midazolam, and the patient was extubated (Figure 3).

**Figure 4:**
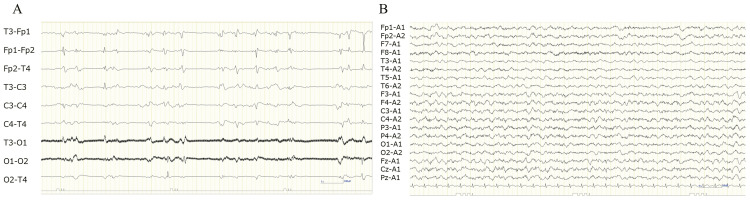
Electroencephalography on days 20 and 41 (A) On day 20, one day after initiating perampanel at 2 mg/kg/day, sporadic sharp waves seen predominantly in the frontal regions during continuous midazolam administration at 0.2 mg/kg/h. (B) On day 41, no sharp waves visible during perampanel administration at 8 mg/kg/day.

The EEG improved as the dose of perampanel was increased (Figure 4).

**Figure 5:**
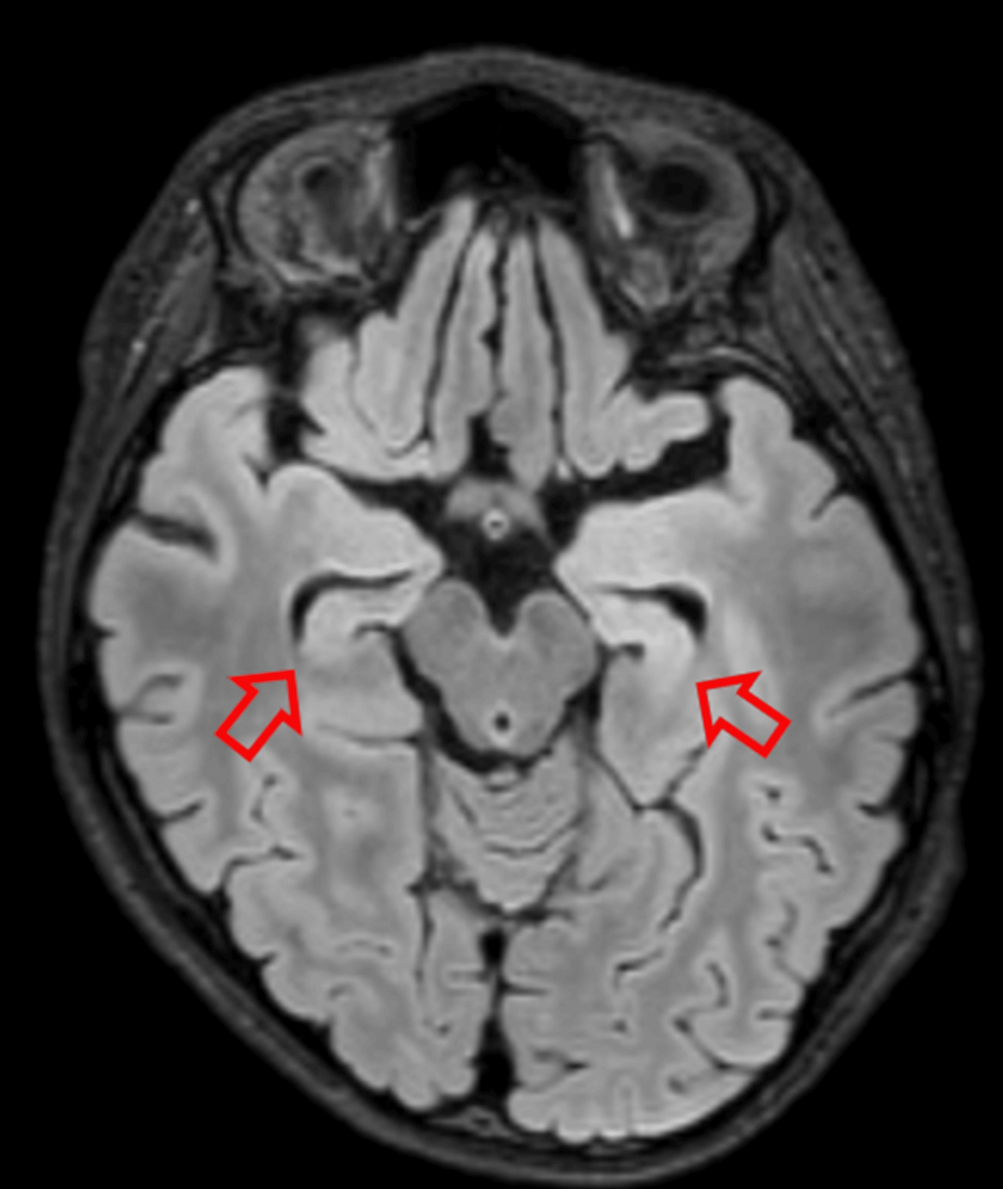
Brain magnetic resonance imaging on day 96 Fluid-attenuated inversion recovery (FLAIR) sequence showing bilateral hippocampal atrophy (red arrows).

Phenobarbital was added to relieve muscle hypertonia but was later discontinued because of drug rashes and liver damage. After intensive care management, clobazam and levetiracetam were added on the 64th day and seizure-free thereafter. MRI showed bilateral hippocampal atrophy (Figure 5).

He had normal motor function but had memory and behavioral issues assessed as Pediatric Cerebral Performance Category (PCPC) 3.

Written informed consent was obtained from the patient’s guardians for participation in the study and publication of the images.

## Discussion

This case report described a 13-year-old boy who received perampanel as part of the treatment strategy for FIRES. Perampanel administration enabled successful weaning from high-dose barbiturate therapy and ventilator support, suggesting its potential role in improving outcomes in FIRES.

We diagnosed this case as FIRES because the patient presented with refractory status epilepticus following a febrile episode and was negative for antineuronal antibodies [6]. The cryptogenic NORSE (C-NORSE) score is known for its ability to differentiate early between C-NORSE, including FIRES, and autoimmune encephalitis with positive antineuronal antibodies [1]. The C-NORSE score comprised six criteria as follows: 1) highly resistant to conventional AED treatment, 2) previously healthy before the onset of status epilepticus, 3) prodromal high fever of unknown origin, 4) absence of prodromal psychobehavioral or memory alterations, 5) absence of sustained orofacial-limb dyskinesia under an unresponsive state, and 6) symmetric DWI or T2/fluid-attenuated inversion recovery (FLAIR) hyperintensities. If five or more of these six criteria are met, the likelihood of detecting antineuronal antibodies is low, and the possibility of cryptogenic NORSE is high. In this case, all the criteria were met.

FIRES often present with refractory epilepsy, and at least 75% of patients require continuous infusion of anesthetics and prolonged burst-suppression coma to control seizures [7]. However, prolonged anesthesia carries the risk of complications, whereas burst-suppression coma is associated with worse neuropsychological outcomes in pediatric FIRES [8]. Perampanel is a noncompetitive antagonist of the AMPA glutamate receptor on postsynaptic neurons and was reported to be effective in controlling focal-onset seizures with or without secondary generalization [9]. In cases of FIRES, the effectiveness of AEDs is considered to be limited, but there have been reports that perampanel may be beneficial [5]. The efficacy of the ketogenic diet for FIRES has already been reported, and its mechanism of action is hypothesized to involve the direct inhibition of postsynaptic excitatory AMPA receptors [10]. Previous studies on pediatric patients with FIRES reported an average of 49 days of ventilator management [8]. In our patient, perampanel administration allowed withdrawal from high-dose barbiturate therapy, which may have enabled earlier liberation from the ventilator.

Acute-phase treatment for NORSE, including FIRES, has been reported in several case reports but has not been clearly demonstrated to be effective [7]. Immunotherapies, such as steroid pulse and intravenous immunoglobulin, are often administered, but their effects are limited [4]. Ketogenic diet therapy is effective for refractory epilepsy in FIRES and is one of the options to be considered [3]. In this case, complications of liver dysfunction and infection associated with high-dose barbiturate therapy and ventilator management made it difficult to implement ketogenic diet therapy. Other treatments, such as cannabidiol and anakinra, have been attempted for off-label use in Japan [11,12]. Perampanel has been suggested to be effective against FIRES [5]. Drowsiness, dizziness, and behavioral problems have been reported as adverse events of perampanel, but serious adverse events are rare [9]. In this case, perampanel was used for refractory epilepsy during high-dose barbiturate therapy, and the patient experienced no adverse events. In patients with NORSE, signal changes and atrophy of the hippocampus have often been reported on MRI [1], likely owing to prolonged convulsive seizures. Moreover, cognitive and behavioral sequelae have been frequently reported in patients with NORSE [3]. This patient presented with behavioral issues, such as inattention and memory impairment but had a disease course that was similar to previously reported cases, indicating potential causes other than perampanel. The use of perampanel in this patient was clinically significant because it reduced the adverse effects of prolonged barbiturate use and complications of ventilator management. The use of perampanel for FIRES has not been widely studied, and the present case may provide evidence of its efficacy and safety.

## Conclusions

This case highlights the potential role of perampanel in managing FIRES by facilitating weaning from high-dose barbiturate therapy and ventilator support, ultimately minimizing the complications associated with prolonged anesthetic use. This approach may help lessen adverse effects and improve overall patient outcomes.

Given that current evidence is limited to case reports and small-scale studies, further research is necessary to evaluate the safety and effectiveness of perampanel in FIRES.
